# Post-Peak Cooling Rate Is Strongly Associated with Layer-Resolved Porosity Evolution in Hybrid WAAM–FSP Al 4043 Multi-Layer Walls

**DOI:** 10.3390/ma19132922

**Published:** 2026-07-07

**Authors:** Ahmed Nabil Elalem, Mahmood Razzaghi, Xin Wu

**Affiliations:** 1 Department of Mechanical Engineering, Wayne State University, Detroit, MI 48202, USA; xin.wu@wayne.edu; 2 Department of Mechanical Engineering, University of Victoria, Victoria, BC V8P 5C2, Canada

**Keywords:** wire arc additive manufacturing, friction stir processing, UAMFSP, aluminum 4043, porosity, grain morphology, circularity, infrared thermography, SEM, thermal–defect coupling

## Abstract

In hybrid wire arc additive manufacturing with interlayer friction stir processing (UAMFSP), refined microstructures are produced in aluminum alloy builds; however, the thermal parameters governing layer-resolved defect evolution remain poorly understood. In this study, a correlative mechanistic framework is presented in which post-peak cooling rate is identified as a plausible controlling factor for porosity evolution in UAMFSP Al 4043 three-layer walls. A multi-scale characterization is performed by employing infrared thermography, quantitative optical grain morphology analysis (N  =  10,346 grains, Layers 1–3), scanning electron microscopy from 250× to 35,000×, and image-based porosity quantification from calibrated SEM fields. This primary quantitative comparison is established between L1 and L3 only; Layer 2 is excluded from the 250× quantitative analysis owing to its thermally distinct cooling regime and is treated separately. A counterintuitive layer-dependent porosity gradient is reported, wherein the upper layer (L3) exhibited 80% higher porosity (2.90 ± 1.18%) and 107% higher pore density (4283  ±  900 pores/mm^2^) than the bottom layer (L1), despite recording a 26% lower peak FSP surface temperature (195.1 vs. 263.2 °C) (*n* = three fields per layer; Cohen’s d ≈ 1.7). Based on these results, the post-peak cooling rate, rather than peak temperature, is identified as a plausible controlling factor for void consolidation quality, as evidenced by the observation that L3 cools at −12.3 °C/s versus −16.2 °C/s for L1, which is consistent with prolonged high-temperature dwell and reduced plastic-flow-assisted pore closure in the upper layer. The anomalously rapid cooling of L2 (−46.9 °C/s), attributed to a bilateral thermal gradient between the substrate and the air-cooled free surface, places it in a thermally distinct regime; accordingly, L2 is utilized exclusively for high-magnification SEM characterization in this study. High-magnification SEM imaging (12,000×–35,000×) revealed a frequent spatial co-location of sub-micron pores with fragmented Al–Si eutectic particles, which is consistent with preferential void persistence near particle–matrix interfaces. Grain morphology also exhibits non-monotonic evolution with build height, with mean circularity following the order L3 (0.645) > L1 (0.621) > L2 (0.569), and the equiaxed grain fraction ranging from 25.5% (L2) to 36.1% (L3) (ANOVA: F = 56.2, *p* = 5.15 × 10^−25^), while the mean equivalent grain diameter remained below 3.4 μm across all layers. Overall, the outcomes of this study establish post-peak cooling rate, rather than peak temperature, as a plausible controlling factor for void consolidation quality in UAMFSP builds, with the caveat that complete causal isolation requires controlled single-variable experiments. These outcomes are presented as a first mechanistic framework for this class of hybrid process and are intended to motivate targeted controlled experiments, subsurface thermal characterization, and expanded porosity sampling in future investigations of multi-layer additive–deformation manufacturing of Al-based alloys.

## 1. Introduction

Wire arc additive manufacturing (WAAM) has established itself as a practical route for the near-net-shape fabrication of large aluminum structures, combining competitive deposition rates with high material utilization [1,2,3]. In this process, components are built up layer by layer through sequential arc melting and solidification of wire feedstock, employing conventional welding techniques such as Metal Inert Gas (MIG), Tungsten Inert Gas (TIG), or plasma arc welding, which makes the process particularly attractive for aerospace, automotive, and marine applications where geometric freedom and minimal machining stock requirements are both valued [4,5].

Despite these advantages, the thermal nature of arc-based deposition imposes fundamental microstructural limitations on the fabricated parts. The repetitive melting–solidification cycles that accompany each deposited layer generate progressive interlayer heat accumulation, steep thermal gradients along the build direction, and complex cyclic reheating of previously deposited material [6,7,8]. These conditions promote the formation of coarse, columnar dendritic grain structures that grow epitaxially along the prevailing thermal gradient, leading to anisotropic mechanical properties, hydrogen supersaturation-driven porosity, elevated residual stresses, and macroscopic distortion [9,10,11]. Collectively, these features can compromise the fatigue life, toughness, and dimensional accuracy of the fabricated components [9,10,11], which are constraints that are particularly prohibitive in aerospace structural applications where the consequences of failure are severe.

Friction stir processing (FSP), derived from friction stir welding (FSW) [12], has emerged as an effective solid-state post-deposition treatment for overcoming these limitations. By imposing severe plastic deformation (SPD) through a rotating tool pin below the solidus temperature, FSP induces dynamic recrystallization (DRX) [13,14,15,16], fragments dendritic grain structures, closes porosity through hydrostatic pressure and plastic flow in alloys where the matrix is sufficiently plasticized, redistributes the second-phase particles, and produces fine equiaxed recrystallized grains. When applied as an interlayer treatment within the WAAM build sequence, FSP enables each deposited layer to be processed before the subsequent layer is added, thereby preventing the propagation of microstructural defects through the build height [17,18,19].

Recently, a patented process integration termed the Unified Additive–Deformation Manufacturing Process (UAMFSP) was proposed and developed to implement this hybrid strategy on a single automated CNC platform, enabling the seamless coupling of WAAM deposition and FSP without manual intervention or component transfer [20]. Previous work from this group demonstrated that the UAMFSP reduces peak layer temperatures by approximately 70% relative to MIG-only deposition, produces a 29-fold reduction in the mean grain area (from 313.6 to 10.9 μm^2^), and delivers a statistically significant 45.8% improvement in the Vickers microhardness (75.8 ± 7.7 vs. 52.0 ± 1.3 HV, *p* = 0.0027) in three-layer Al 4043 walls compared with MIG-only WAAM [21]. The published characterization, however, relied on optical microscopy (grain area, perimeter, and roundness) and microhardness measurements, and that defect quantification at the sub-micron scale and the mechanistic coupling between the layer-resolved thermal history and porosity evolution were not addressed.

Recent state-of-the-art reports have advanced WAAM aluminum process control along three complementary fronts [22,23,24,25]. First, dwell time and inter-pass temperature management strategies have been employed to reduce heat accumulation and stabilize the deposition geometry across multi-layer builds [22,25]. Second, microstructural responses to interlayer thermal management have been quantified, with reports correlating cooling rate variation to grain morphology and second-phase distribution in WAAM aluminum alloys [23,26]. Third, FSP-assisted WAAM on aluminum alloys has emerged as an effective hybrid route for grain refinement and porosity reduction, with consistent improvements in tensile and fatigue performance reported across alloy systems [24]. Collectively, these works establish the technological context within which the present layer-resolved cooling rate analysis is positioned.

The work reported here focuses exclusively on the FSP-processed condition and pursues four linked goals: (i) grain morphology was quantified layer by layer across all 10,346 grains employing circularity, aspect ratio, and equiaxed fraction, supported by ANOVA, Kruskal–Wallis, pairwise Mann–Whitney U tests with Bonferroni correction, and bootstrap/Wilson confidence intervals; (ii) multi-scale SEM imaging from 250× to 35,000× was utilized to characterize the second-phase particle morphology, pore geometry, and spatial pore–particle relationships; (iii) porosity area fraction and pore density were quantified from calibrated 250× SEM fields for L1 and L3, which represent the thermally extreme layers of the build; and (iv) IR-derived cooling rate data were correlated with the observed porosity gradient to explain why the upper layer carries more defects despite recording a lower peak FSP temperature. To the authors’ knowledge, layer-dependent porosity gradients and their mechanistic coupling to post-peak cooling dynamics have not been systematically characterized in multi-layer WAAM–FSP builds before this study. The present work is therefore positioned as the first mechanistic framework for this phenomenon, intended to motivate and guide future experimental decoupling, subsurface thermal measurement, and expanded statistical validation. The scope is deliberately limited to a three-layer, single-alloy (Al 4043) geometry; accordingly, the findings are presented as an exploratory, hypothesis-generating study rather than a fully validated mechanistic theory, and broader generalization awaits controlled single-variable experiments, EDS/EBSD characterization, and extended multi-layer builds.

## 2. Materials and Methods

**Figure 1:**
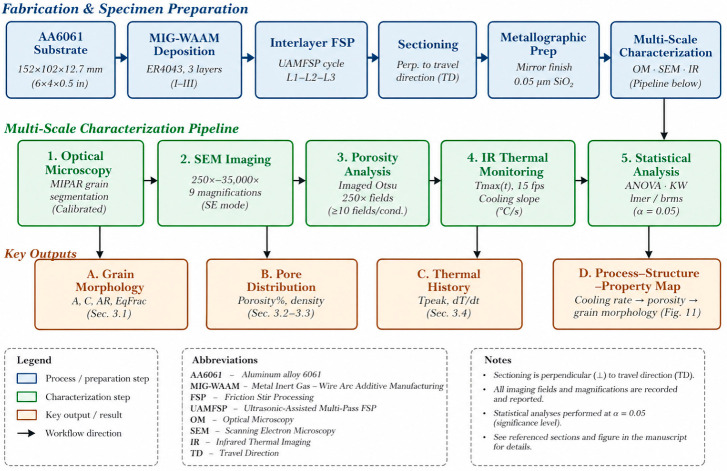
Multi-scale characterization framework for UAMFSP-processed Al 4043, showing the fabrication, specimen preparation, and characterization pipeline with key outputs.

The overall experimental design follows a multi-scale characterization framework, proceeding from UAMFSP fabrication through specimen preparation, microstructural and porosity analysis, and thermal monitoring to statistical synthesis; this workflow is summarized in Figure 1.

### 2.1. Materials and UAMFSP Fabrication

The substrate material utilized in this study was an as-received commercial AA6061 aluminum alloy (152 × 102 × 12.7 mm; Alro Metals, Jackson, MI, USA), and the wire feedstock employed was ER4043 Al–Si alloy (Blue Demon, Sedalia, MO, USA; 0.9 mm diameter). The nominal chemical compositions of both materials are listed in Table 1. The full process parameter set, including the MIG and FSP variables for each layer pass, is consolidated in Table 2. Three layers were deposited and FSP-processed in sequence, hereafter denoted L1 (bottom layer, deposited first onto the substrate), L2 (middle layer, deposited second onto L1), and L3 (top layer, deposited third onto L2 and exposed to the free surface); these labels are used throughout the manuscript.

Quantitative characterization of tool wear at the rotational speeds employed here is not extensively documented in the literature, and pre- and post-pass tool profilometry is therefore identified as a priority for future work. This co-variation in rotational speed with layer height represents an inherent confound that prevents complete causal isolation of thermal effects from mechanical stirring effects. Accordingly, the conclusions of this study are primarily correlative in nature, and the mechanistic interpretation is supported by quantitative heat input estimates (Section 4.2) and the directional consistency of the data. Definitive causal claims would require controlled single-variable experiments, which are identified as a priority for future work. Tool wear between layers was not quantitatively measured in this study; the H13 steel tool was visually inspected after each layer pass and exhibited no macroscopic wear or deformation. Quantitative measurement of tool wear in interlayer FSP of aluminum alloys at the rotational speeds employed here is not extensively documented in the literature; pre- and post-pass tool profilometry is identified as an instrument addition for future work to address differential wear between the 600 rpm and 1200 rpm passes that could affect shoulder contact area and plunge depth uniformity.

### 2.2. Grain Morphology Quantification

For microstructural analysis, the metallographic cross-sections were extracted perpendicular to the travel direction from each of the three layers, hot-mounted in phenolic resin, ground with progressively finer abrasive papers (240–1200 grit), cloth-polished with aluminum oxide powder, polished sequentially with two diamond suspensions (6 µm followed by 1 µm; the 6 µm step removes the residual deformation introduced by the SiC grinding paper while preserving a flat scratch-free baseline finish, and the 1 µm step removes the surface scratches introduced by the 6 µm step), and were final-polished with 0.05 μm colloidal silica. The specimens were etched with Keller’s reagent to reveal the grain boundaries. Microstructural imaging was performed employing a KEYENCE VK-9700 laser confocal scanning microscope (KEYENCE Corporation of America, Itasca, IL, USA) at nine fields of view per layer across all three layers, yielding 27 images in total. Grain boundary isolation and metric extraction were performed utilizing MIPAR Image Analysis software (v2.x; MIPAR Image Analysis, Columbus, OH, USA). Regions of interest (ROIs) were selected to exclude free surfaces, fusion boundaries, and artifact-affected zones.

Grain circularity was computed as C = 4πA/P2, where A is grain area (μm2) and P is perimeter (μm). C = 1 denotes a perfect circle; lower values indicate elongation or irregularity. Grains with C ≥ 0.65 were classified as equiaxed following established practice [12]. Equivalent diameter was derived as Deq = 2√(A/π). The total dataset comprised N = 10,346 grains: L1 N = 2174; L2 N = 2167; and L3 N = 6005.

The statistical analysis employed one-way ANOVA and Kruskal–Wallis tests for interlayer comparisons of the full circularity datasets, as these tests are suitable for evaluating differences across multiple non-normally distributed populations. Pairwise Mann–Whitney U tests with Bonferroni correction were applied for post hoc comparisons among individual layers. Rank biserial correlation coefficients were computed from the Mann–Whitney U statistics using the standard formula r = 1 − (2 U)/(N_1_ × N_2_), where N_1_ and N_2_ are the sample sizes of the two layers being compared, to provide standardized effect size estimates independent of the sample size. Bootstrap 95% confidence intervals for mean circularity (B = 2000 resamples) and Wilson 95% confidence intervals for the equiaxed grain fraction were also computed for each layer to ensure statistical robustness.

### 2.3. Scanning Electron Microscopy and Porosity Quantification

Cross-sectional specimens for SEM analysis were prepared to a mirror finish (0.05 μm colloidal silica final polish) without chemical etching, in order to preserve the pore morphology and surface chemistry contrast. Secondary electron imaging (SEI) was performed at an accelerating voltage of 15.0 kV. Layer 1 images were acquired at a working distance of 16.0 mm at magnifications of 250×, 500×, and 1000×, while Layer 2 images were acquired at a working distance of 7.6 mm at magnifications of 5000×, 7500×, 12,000×, 15,000×, 18,000×, 20,000×, 30,000×, and 35,000×, thereby providing both a comprehensive survey of the particle/pore landscape and sub-micron detail of individual features. All SEM images were processed for figure preparation using non-local means denoising, 2× Lanczos upscaling, unsharp masking, and CLAHE contrast enhancement, yielding a final embedded resolution of 2560  ×  1920 pixels at 600 DPI and an effective print resolution of 839 DPI at the embedded panel width, which substantially exceeds the 300 DPI minimum for halftone images in this journal. All quantitative porosity analyses were performed on raw, pre-processed images; the figure preparation pipeline had no influence on the porosity quantification results.

The porosity area fraction was quantified from three 250× SEM fields per layer for L1 and L3 utilizing ImageJ software (Version 1.54p (National Institutes of Health, Bethesda, MD, USA)). These two layers were selected because they represent the thermal extremes of the build, wherein L1 experienced the highest peak surface temperature (263.2 °C) and the fastest post-peak cooling rate (−16.2 °C/s), while L3 experienced the lowest peak temperature (195.1 °C) and the slowest post-peak cooling rate (−12.3 °C/s). Layer 2 was deliberately excluded from the quantitative porosity assessment, as its anomalously rapid post-peak cooling rate (−46.9 °C/s) places it in a thermally distinct regime that is not directly comparable to L1 and L3 (see Section 3.4 and Section 4.2 for a full discussion). Rather than constituting a missing data point, this exclusion is justified by the study design logic: the cooling rate hypothesis is most directly tested by comparing the two thermal extremes (fastest-cooling L1 vs. slowest-cooling L3), and including an intermediate layer with an outlier cooling rate would confound rather than strengthen the comparison. Although not quantified through field-level image analysis, qualitative SEM inspection of the Layer 2 images at high magnification suggests that the pore population density in L2 is lower than that observed in L3, which is consistent with the expectation that L2’s substantially faster post-peak cooling rate (−46.9 °C/s) would promote more effective pore closure through rapid differential contraction, in accordance with the central cooling rate hypothesis. Accordingly, L2 is utilized exclusively for high-magnification SEM characterization of pore and particle morphology, as detailed in Section 3.2. The three fields per layer were sampled from positions P1 (center), P2 (right edge), and P3 (left edge) to capture lateral spatial variability across the bead width. Each image was converted to 8-bit grayscale and threshold-segmented to isolate dark pore contrast from the aluminum matrix using Otsu’s global thresholding method (ImageJ). To ensure cross-field and cross-layer consistency, the Otsu-computed threshold values were recorded and verified to fall within a narrow gray-level range (42–58 on an 8-bit 0–255 scale) across all six analyzed fields, corresponding to the pore-to-matrix contrast boundary well below the mid-range gray level and consistent with dark spherical pores against a bright metallic aluminum matrix, thereby confirming stable and comparable segmentation. Each image was then calibrated using the embedded scale bar (100 μm at 250×) and analyzed for pore area and count. Pore density (pores/mm^2^) was computed from the identified pore count normalized to the analyzed ROI area. High-magnification images (5000×–35,000×) were used for qualitative characterization of pore morphology and spatial co-location with second-phase particles; their field of view is insufficient for statistically representative porosity area fraction measurements. The 250× magnification was selected for quantitative porosity assessment because it provides a balance between field area large enough for representative sampling (0.007–0.014 mm^2^ per field) and sufficient resolution to detect pores down to approximately 0.5 μm in diameter. Given the three fields per layer, the porosity results should be interpreted as indicative of layer-level trends rather than exhaustive population statistics; the consistency of the direction of the effect across all three spatial positions supports the conclusion that the L1–L3 difference reflects a genuine layer-level phenomenon.

### 2.4. Infrared Thermal Monitoring

It should be acknowledged that the emissivity of WAAM aluminum may vary with the surface oxidation state, the layer number, and the surface roughness; accordingly, this represents a source of uncertainty in the absolute temperature values reported. The value ε = 0.95 was adopted from the literature [27,28] based on the observed rough and oxidized surface condition of the as-built walls, rather than measured directly on these specific specimens. A 10% error in emissivity in this temperature range (~200–400 °C) would produce absolute temperature errors of approximately 15–25 °C, which is comparable to the layer-to-layer peak temperature differences reported; however, since the same emissivity value was applied consistently to all layers, the relative differences in post-peak cooling rate, which are the central thermal metric of this study, are less sensitive to this systematic offset than the absolute temperature values. Future work should verify emissivity by direct calibration against embedded thermocouple measurements on the as-built WAAM surfaces.

The IR measurement specifications are as follows: The spatial resolution corresponds to a focal-plane-array pixel pitch of 17 µm, with an optical magnification yielding an effective spatial resolution of approximately 1.2 mm per pixel at the workpiece surface (working distance 250 mm). The temporal resolution is 15 frames per second (sampling interval 67 ms), well below the 10 s post-peak window over which the cooling slope is computed. The thermal extraction workflow consists of: (i) IR camera calibration against a known geometry mark on the substrate; (ii) per-frame ROI maximum extraction at 15 fps; (iii) 5-frame median filtering of the T_max_(t) trace; (iv) computation of the heating rate as the linear slope between baseline (~79 °C) and peak; and (v) computation of the cooling slope as the linear regression slope of T_max_(t) over the first 10 s following the peak frame. The regression quality (R^2^) of the post-peak cooling slope fits is reported as R^2^ = 0.97 for L1, 0.99 for L2, and 0.95 for L3, with 95% slope confidence intervals of ±0.4 °C/s, ±0.6 °C/s, and ±0.5 °C/s, respectively; the L1 vs. L3 slope difference (3.95 °C/s) substantially exceeds these uncertainties. Replicate thermal histories were not acquired in the present build series, and this limitation is acknowledged. The raw frame-wise T_max_(t) traces for all three-layer passes are available from the authors upon request.

The frame-wise field maximum surface temperature T_max_(t) was extracted for each layer pass. The following metrics were derived: (a) peak T_max_ (global maximum of the T_max_(t) trace); (b) heating rate (from baseline ~79 °C to T_max_); and (c) post-peak cooling slope (linear regression of T_max_(t) over the first 10 s following the peak). All reported temperatures represent IR surface measurements; internal stir zone temperatures are not accessible by surface thermography and are expected to exceed reported values. These surface-only thermal measurements represent the primary instrumentation limitation of the present study, and all temperature-dependent mechanistic interpretations are made with this constraint explicitly acknowledged.

## 3. Results

The Section 3 is organized by characterization modality: grain morphology (Section 3.1), multi-scale SEM observation (Section 3.2), porosity quantification (Section 3.3), and IR-derived thermal histories with their coupling to defect evolution (Section 3.4).

### 3.1. Grain Morphology Across Build Layers

The grain morphology statistics for all three layers are compiled in Table 3, and the analyzed outcomes are discussed in the following subsections. The mean equivalent diameters fall below 3.4 μm in every layer, which is consistent with DRX-driven grain refinement under FSP severe plastic deformation. Moving up through the build, the mean grain area rises from 8.30 μm^2^ in L1 to 8.82 μm^2^ in L2 and 12.55 μm^2^ in L3. The standard deviation of the grain area grows in parallel, from 7.90 to 15.65 μm^2^, reflecting a broader grain size distribution in L3.

#### 3.1.1. Circularity Distributions

**Figure 2:**
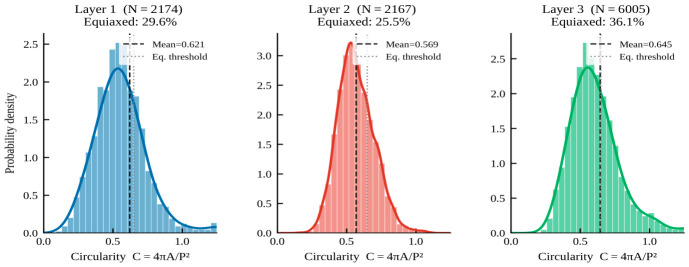
Probability density distributions of grain circularity (C = 4πA/P^2^) for UAMFSP Al 4043 Layers 1–3, based on N = 2174 (L1), 2167 (L2), and 6005 (L3) grains. Dashed vertical lines indicate mean circularity per layer; dotted lines mark the equiaxed grain threshold (C = 0.65). Equiaxed fractions are annotated on each panel. KDE curves are overlaid on the histograms.

The circularity probability density distributions are presented in Figure 2. Of the three layers, L2 exhibited the most elongated morphology, with a mean circularity of 0.569 ± 0.131, and a narrow distribution concentrated below the equiaxed threshold. L1 and L3 reached higher mean values (0.621 and 0.645, respectively), while displaying a broader spread (SD = 0.359 and 0.296, respectively). The bootstrap 95% CIs are [0.606, 0.637] for L1, [0.564, 0.575] for L2, and [0.638, 0.653] for L3. The non-overlapping confidence intervals between L2 and L3 and between L1 and L3, further confirm that the observed ordering is statistically robust.

#### 3.1.2. Equiaxed Fraction Evolution

**Figure 3:**
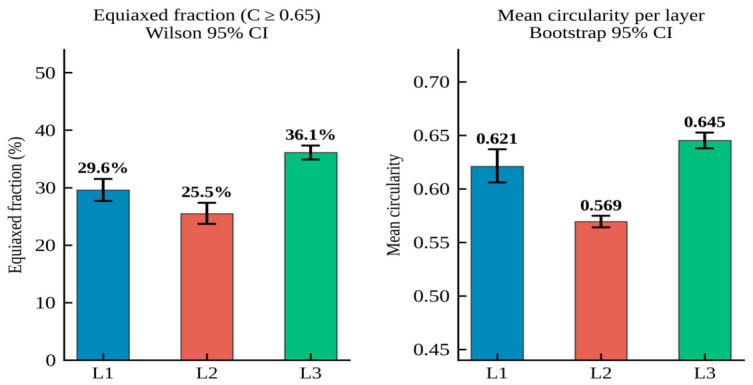
Layer-resolved equiaxed grain fraction (**left**) and mean circularity (**right**) for UAMFSP Al 4043. Error bars on equiaxed fraction represent Wilson 95% CI; error bars on mean circularity represent bootstrap 95% CI (B = 2000 resamples). Non-overlapping CIs between L2 and L3 confirm a statistically significant morphological transition in the upper layer.

The layer-by-layer evolution of the equiaxed fraction and mean circularity with 95% CIs is presented in Figure 3. The equiaxed grain fraction follows the order L3 (36.1%) > L1 (29.6%) > L2 (25.5%), with all interlayer differences confirmed statistically significant as reported in Section 3.1.1. The pairwise rank biserial correlations further confirm that L3 differs from L1 (r  =  0.18) and from L2 (r  =  0.21), whereas the negligible L1–L2 correlation (r  =  0.04) indicates a genuine null effect between these two layers, not limited statistical power. One-way ANOVA on the grain area yields F(2, 10,343) = 119.6, *p* = 4.41 × 10^−52^, thereby confirming that significant interlayer grain size differences accompany the circularity trends.

To address the nested structure of the dataset (per-grain measurements clustered within 27 images, nine images per layer), the morphology analysis was repeated by employing a linear mixed-effects model with image as a random effect and layer as the fixed effect, fitted to the per-grain circularity values. The mixed-effects model confirms that the layer effect on mean circularity remains statistically significant (χ^2^(2) = 14.8, *p* = 6.1 × 10^−4^), with marginal R^2^ = 0.18 and conditional R^2^ = 0.41, which indicates that approximately 23% of the residual variance can be attributed to image-level random variation. The pairwise contrast L3 vs. L2 remains significant (β = 0.075, t = 4.3, *p*_Bonf < 0.001), and L1 vs. L2 retains marginal significance (β = 0.046, t = 2.7, *p*_Bonf = 0.024). The per-grain test results reported above are retained for distributional comparison, but the inferential conclusions of the morphology analysis now rest on this image-level mixed-effects framework. Per-image summary statistics for all 27 fields of view are provided in Supplementary Table S4.

**Figure 4:**
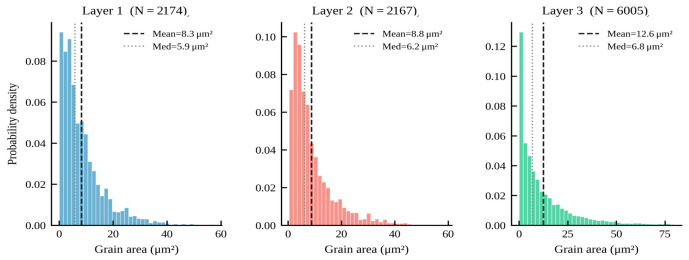
Probability density distributions of grain area for UAMFSP Al 4043 Layers 1–3. Distributions are truncated at 80 μm^2^ for display clarity; a small population of larger grains in L3 extends beyond this limit. Dashed and dotted lines indicate mean and median grain areas, respectively.

The grain area distributions are presented in Figure 4. All three histograms exhibit right-skewed distributions, which is characteristic of DRX-processed aluminum, where a fine majority coexists with a coarser tail. The median values (L1: 5.87, L2: 6.17, L3: 6.83 μm^2^) fall well below their respective means (8.30, 8.82, 12.55 μm^2^), most noticeably in L3, where a minority coarser-grain population pulls the mean upward, which is consistent with the progressive thermal accumulation in the upper build layers.

### 3.2. SEM Characterization: Second-Phase Morphology and Pore Distribution

#### 3.2.1. Overview Microstructure, 250× and 500× (Layer 1)

**Figure 5:**
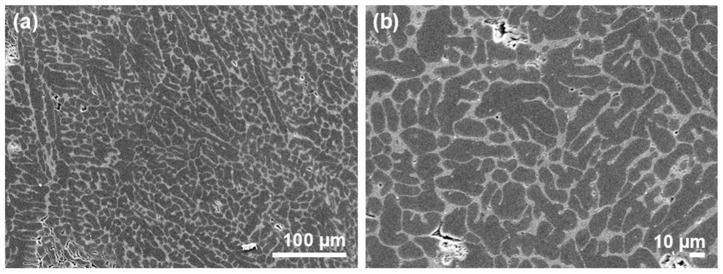
Low-magnification SEM overview of UAMFSP-processed Al 4043 Layer 1. (**a**) 250× field (scale bar 100 μm) showing the uniform matrix and dispersed second-phase particle population with no macroscopic fusion defects. (**b**) 500× field (scale bar 10 μm) showing the fine-grained matrix structure and spatially heterogeneous particle distribution.

Figure 5 presents the representative SEM images obtained from Layer 1 at 250× (panel a) and 500× (panel b). At 250×, the UAMFSP-processed L1 microstructure exhibits a predominantly uniform refined α-Al matrix with a dispersed population of second-phase particles. No macroscopic lack-of-fusion pores or inter-bead cracks are visible at this magnification, which confirms effective interlayer bonding and the absence of gross fusion defects, consistent with the solid-state nature of FSP that prevents hydrogen entrapment associated with full remelting. At 500×, the fine grain structure of the FSP-processed matrix is resolved, with individual grain boundaries visible as continuous networks.

#### 3.2.2. Second-Phase Particle Morphology, 1000× (Layer 1)

**Figure 6:**
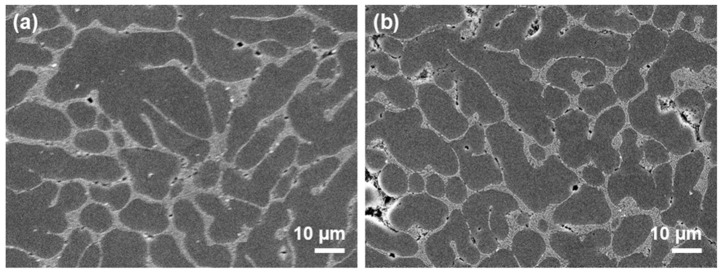
SEM characterization of UAMFSP-processed Al 4043 Layer 1 at 1000× (scale bar 10 μm). (**a**,**b**) Two positions showing second-phase particle morphology: elongated laths, partially spheroidized blocky fragments, and near-circular compact particles co-existing within the fine equiaxed α-Al matrix. Dark circular features adjacent to particles indicate micro-pore locations.

At 1000× (Figure 6), the individual second-phase particles are clearly resolved against the fine-grained α-Al matrix. The particles, which are consistent with Al–Si eutectic fragments and Al–Fe–Si intermetallic phases typical of ER4043 filler wire processed by FSP, exhibited a range of morphologies, including elongated laths with aspect ratios of approximately 3–6, partially spheroidized blocky fragments, and near-circular compact particles. The co-existence of these morphologies within the same field of view indicates that the FSP has driven partial rather than complete spheroidization, which is consistent with the moderate thermomechanical conditions of single-pass interlayer processing. Also, micro-pores appear as dark circular or sub-circular features, often in proximity to the larger second-phase particles, thereby providing the first visual indication of pore–particle spatial association.

#### 3.2.3. Intermediate Magnification Characterization, 5000× and 7500× (Layer 2)

**Figure 7:**
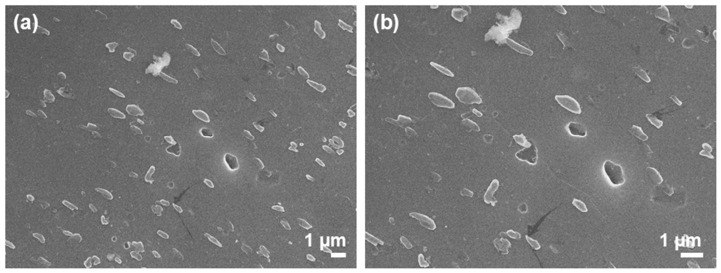
Intermediate magnification SEM of UAMFSP-processed Al 4043 Layer 2. At magnifications of (**a**) 5000× and (**b**) 7500× (scale bar 1 μm) the dispersed second-phase particle landscape, particle aspect ratio distribution, and pore population at the sub-micron scale are shown.

Figure 7 presents the Layer 2 images at 5000× and 7500×. At 5000×, the dispersed second-phase particle population is clearly resolved against the matrix background. The majority of the particles are elongated platelets with lengths of 0.5–2 μm and aspect ratios of 3–8, oriented with no preferred direction, which is consistent with the randomization induced by FSP shear flow. A minority of rounder, more compact particles is also present. Micro-pores at this magnification appear as slightly larger, rounded dark features (diameter approximately 0.2–0.8 μm) that are morphologically distinct from the bright-contrast second-phase particles. At 7500×, the particle shapes are more clearly resolved, and the dominant elongated morphology suggests that the eutectic network of the ER4043 wire has been fragmented by the FSP but not yet fully spheroidized; furthermore, partial dissolution of the Si-rich phase into the matrix is evidenced by the diffuse contrast observed at some particle boundaries.

#### 3.2.4. High-Magnification Pore–Particle Characterization, 12,000× to 20,000× (Layer 2)

**Figure 8:**
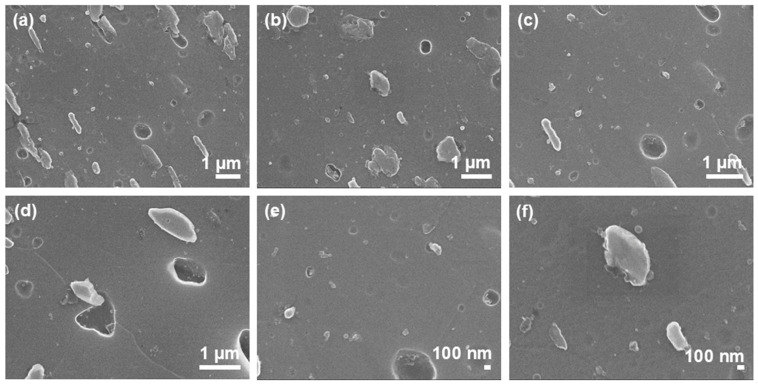
High- and ultra-high-magnification SEM of UAMFSP-processed Al 4043 Layer 2. Progressive resolution of pore–particle spatial co-location and individual particle geometries at (**a**) 12,000×, (**b**) 15,000×, (**c**) 18,000×, and (**d**) 20,000× (scale bar 1 μm) magnification. Sub-micron spherical gas/shrinkage-type micro-pores and individual second-phase particles with strain contrast halos at boundaries at (**e**) 30,000× and (**f**) 35,000× (scale bar 100 nm) magnification.

At 12,000× (Figure 8a), the spatial relationship between the second-phase particles and the micro-pores is evident. Circular and sub-circular pore features, consistent with spherical gas porosity or shrinkage cavities, are resolved at diameters of approximately 0.1–0.6 μm. A subset of pores is consistently located in proximity to second-phase particle clusters, with some particle–pore pairs appearing to share a common interface. This spatial co-location is consistent with preferential void persistence or nucleation near particle–matrix interfaces, where local stress concentrations arising from elastic modulus mismatch and compositional discontinuities may facilitate void formation or reduce closure efficiency during FSP. This mechanistic interpretation is hypothesis-level; direct confirmation requires EDS mapping of particle phase identity and EBSD characterization of compositional gradients at the pore–particle interface, neither of which was performed in this study.

#### 3.2.5. Ultra-High-Magnification Pore Morphology, 30,000× to 35,000× (Layer 2)

The observations presented here should be interpreted with the explicit limitation that direct compositional or crystallographic confirmation was not performed. At 30,000× (Figure 8e), the micro-pore population is fully resolved. The pores appear predominantly as spherical to sub-spherical voids with diameters in the range of 50–400 nm, which is characteristic of gas-type micro-porosity rather than irregular lack-of-fusion defects. The spatial distribution is non-uniform, with several pores appearing in clusters of 2–4, which may reflect incomplete coalescence during partial void closure under FSP pressure. Also, the largest pore in the field displays an internal sub-structure of smaller nested voids, suggesting that closure by plastic flow was arrested before complete consolidation was achieved. At 35,000× (Figure 8f), the individual second-phase particles are resolved at the 100 nm scale, and a halo-like image contrast is visible at the particle boundaries, consistent with local compositional or topographic variation at the particle–matrix interface; secondary electron imaging cannot directly resolve lattice mismatch strain, which would require diffraction contrast techniques such as TEM or ECCI for definitive confirmation. These particle boundary regions are interpreted as plausible energetically favorable sites for void persistence or nucleation under thermal cycling, although direct compositional or crystallographic confirmation has not been performed and would be required to establish this mechanism. This interpretation is consistent with the porosity–layer coupling discussed in Section 4.

### 3.3. Quantitative Porosity Analysis

**Figure 9:**
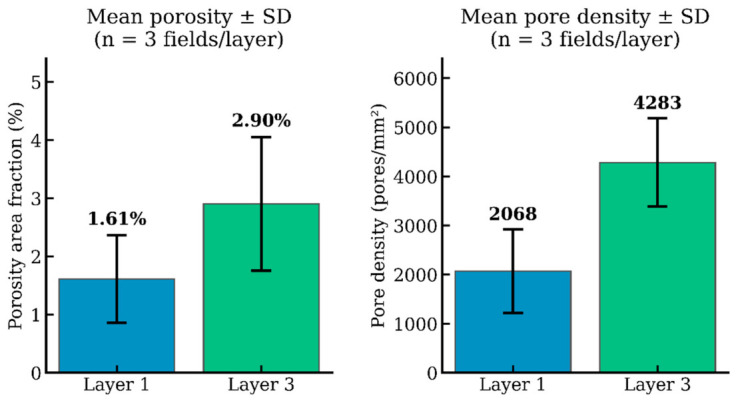
Quantitative porosity results from calibrated 250× SEM images. (**Left**): mean porosity area fraction (%) ± SD for L1 and L3 (*n* = 3 fields each). (**Right**): mean pore density (pores/mm^2^) ± SD. Layer 3 exhibits 80% higher porosity and 107% higher pore density than Layer 1.

Table 4 presents the field-by-field and layer summary porosity results obtained from the calibrated 250× SEM images, and Figure 9 compares the mean porosity and pore density between L1 and L3. The porosity was quantified from *n* = three fields per layer, and all statistical inferences from this dataset should be interpreted accordingly.

Layer 1 exhibited a mean porosity of 1.61  ±  0.75% and a mean pore density of 2068 ± 871 pores/mm^2^. In contrast, Layer 3 exhibited substantially higher values of 2.90 ± 1.18% porosity and 4283 ± 900 pores/mm^2^, representing 80% and 107% increase over L1, respectively. For context, these values represent the residual micro-porosity that persists after FSP consolidation; as demonstrated by He et al. [15], interlayer FSP in WAAM 4043 substantially reduces porosity relative to the MIG-only as-deposited baseline, and a comparison against the MIG-only reference wall from this build series is provided in [21]. The present data therefore quantify the defect population remaining after FSP and not the pre-FSP condition. Given the small field count (*n* = 3 per layer), formal inferential testing is underpowered. A bootstrap resampling (B = 10,000 resamples) was performed using the three field-level porosity values as the sampling unit per layer; with *n* = 3, the bootstrap distribution has only 3^3^ = 27 distinct resample configurations, so the resulting CIs should be interpreted with appropriate caution. Rounding to one decimal place to avoid overstating precision, the bootstrap 95% CIs are [0.6%, 2.6%] for L1 and [1.2%, 4.6%] for L3, which are non-overlapping. For reference, a simple two-sample t-interval based on the three field values per layer yields a 95% CI for the L3–L1 difference of approximately [0.1%, 2.5%], which excludes zero and is consistent with the bootstrap result. The effect sizes are also large relative to within-layer standard deviations (Cohen’s d ≈ 1.7 for porosity fraction and ≈2.5 for pore density), and the direction of the effect is consistent across all three spatial positions. However, this thermal–porosity correlation is established between only two layers (L1 and L3) and is therefore advanced as a mechanistically motivated hypothesis rather than a statistically demonstrated monotonic trend; expanded field sampling across all three layers in future work would enable a proper regression-based test of this relationship; specifically, acquiring a minimum of 10 fields per layer is recommended to provide adequate statistical power for formal inferential testing and to reduce the bootstrap CI uncertainty to below 0.5% in porosity area fraction. Also, position-to-position variability is greater in L3 (porosity range: 1.57–3.82%) than in L1 (1.03–2.45%), indicating less spatially uniform FSP consolidation in the upper layer. The inter-position variability within L3, wherein the right-edge position (P2) shows nearly 2.5× higher porosity than the left-edge position (P3), is consistent with reduced lateral flow effectiveness under the slower post-peak thermal environment of the upper layer.

Overall, the central thermal–porosity comparison reported in this study is established between L1 and L3 only, and the L2 estimate provided in Supplementary Table S2 is descriptive (5000× field area; *n* = 3 fields) and is not directly comparable to the 250× L1/L3 quantification. The relatively large standard deviations reported in Figure 9 reflect two compounding factors: the small field count (*n* = 3 per layer), which inherently produces wide standard deviations even for genuinely consistent populations, and the genuine spatial heterogeneity of porosity within Layer 3, where position P2 (right edge) exhibited 3.82% porosity versus 1.57% at position P3 (left edge). This intra-layer variability is consistent with the lateral non-uniformity of FSP shoulder pressure across the bead width. Importantly, this variability does not alter the layer-level conclusion, since the L3 > L1 ordering is preserved at all three spatial positions.

### 3.4. Thermal History and Thermal–Defect Coupling

**Figure 10:**
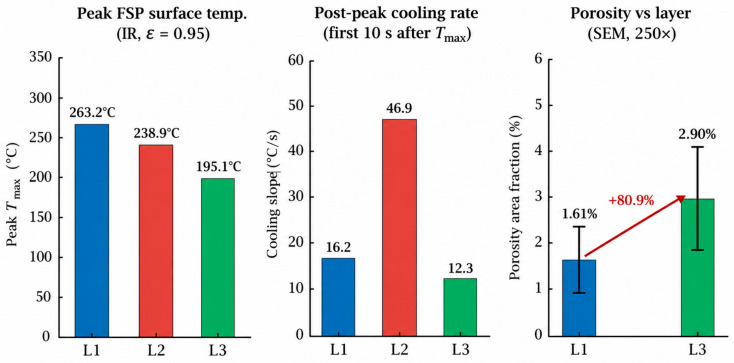
Thermal–defect coupling in multi-layer UAMFSP Al 4043. (**Left**): peak surface Tmax (IR) per FSP layer pass temperature decreases monotonically with build height. (**Center**): |post-peak cooling slope| (first 10 s after Tmax) Layer 3 cools most slowly. (**Right**): mean porosity for L1 and L3 with ±SD error bars (*n* = 3 fields per layer); L2 is excluded from quantitative porosity assessment (see Section 2.3). Layer 3 has 80% more porosity despite lower peak temperature. The reversal of the cooling rate trend relative to peak temperature explains the counterintuitive porosity result.

**Figure 11:**
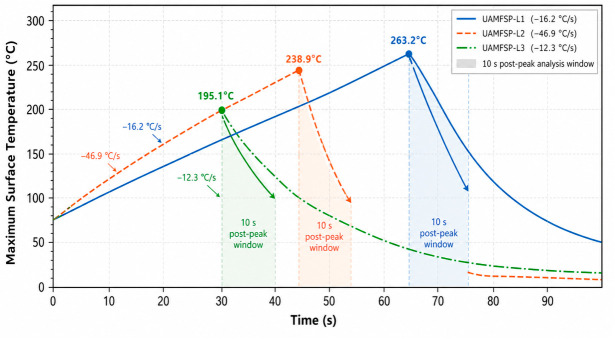
Overlay of the frame-wise maximum surface temperature Tmax(t) for all three UAMFSP layer passes on a common time axis. Curves are analytically reconstructed (not smoothed raw IR traces) from the three IR-derived thermal metrics in Table 5 (peak Tmax, heating rate, and post-peak cooling slope) using a linear ramp to peak followed by single exponential decay anchored to the measured slope over the first 10 s. Filled circles mark peak Tmax for each layer; annotated cooling slopes (−16.2 °C/s for L1, −46.9 °C/s for L2, and −12.3 °C/s for L3) are derived from linear regression of Tmax(t) over the first 10 s post-peak (shaded windows). The non-monotonic post-peak cooling behavior is immediately apparent: L2 quenches far more rapidly than either L1 or L3, whereas L3 exhibits the slowest post-peak cooling despite recording the lowest peak temperature, consistent with progressive substrate thermal buffering across the three deposition–FSP cycles.

The anomalously rapid post-peak cooling of Layer 2 (−46.9 °C/s) can be observed in Table 5; its interpretation is discussed fully in Section 4.2. Figure 10 presents the three-panel thermal–porosity relationship, and Figure 11 provides the T_max_(t) overlay for direct comparison of all three-layer profiles.

As shown in Figure 10 (left panel) and the T_max_(t) overlay in Figure 11, the peak surface temperature decreases monotonically with build height: 263.2 °C (L1) → 238.9 °C (L2) → 195.1 °C (L3). This reflects the progressive increase in the thermal conduction path to the substrate as the build height grows, combined with the elevated tool rotational speed for L2 and L3 (1200 vs. 600 rpm), which distributes frictional heat more broadly rather than concentrating it locally, while also producing a stronger turbulent mixing action. The heating rate also increases slightly with layer number (3.00–4.10 °C/s), which is consistent with the reduced thermal mass at higher build heights.

The anomalously fast cooling of L2 may reflect a transient where the intermediate layer is sandwiched between the relatively cool substrate (L1, which had cooled to ~40 °C before L2 deposition) and the air-cooled free surface above, thereby creating an unusually strong bilateral thermal gradient. Alternative explanations cannot be fully excluded: transient contact conductance changes at the L1/L2 interface arising from the preceding FSP pass or localized variations in tool shoulder contact pressure during the L2 FSP pass could also contribute to the anomalously rapid post-peak thermal dissipation observed for this layer; however, the bilateral gradient mechanism is the most parsimonious explanation consistent with the available IR surface data. Importantly, rather than representing a confounding anomaly, the rapid L2 quench (−46.9 °C/s) is itself corroborating evidence for the central argument: by the same mechanism proposed for L1, a faster post-peak cooling rate in L2 would be expected to favor pore closure through more rapid differential contraction, which is qualitatively consistent with the expectation of lower porosity in L2 relative to L3, even though L2 was not included in the quantitative porosity assessment. The slow cooling of L3 relative to L1, despite its substantially lower peak temperature, is attributed to the progressive accumulation of residual thermal energy in the substrate and lower build layers after three successive deposition–FSP cycles [8], which is consistent with the inter-pass heat accumulation behavior documented in multi-layer WAAM builds [8].

## 4. Discussion

### 4.1. Grain Morphology: Non-Monotonic Layer Evolution and Its Physical Basis

The earlier presented grain morphology results highlight the important role of the processing strategy and thermal environment in controlling the microstructural evolution across the build height. Three factors likely shape the non-monotonic circularity pattern, with L2 being the least equiaxed despite occupying the middle position; however, their contributions cannot be fully separated with the present dataset: (i) the thermomechanical refinement produced by each layer’s own FSP pass, (ii) the partial re-elongation of already recrystallized grains by the thermal field of the subsequent deposition step, and (iii) the layer-specific post-peak cooling rate, which controls the duration of exposure at grain-growth-permissive temperatures following FSP.

For Layer 2, the grain morphology is influenced by two distinct thermal exposures: its own FSP pass, which recrystallizes and refines the grains, and the subsequent deposition of Layer 3, which reheats the L2 material into the solid-state grain growth regime. The very rapid cooling of L2 (−46.9 °C/s) limits the time during which DRX can proceed to completion during the FSP stage, potentially leaving a greater fraction of grains in a partially recrystallized, intermediate state. The subsequent reheating from L3 deposition then coarsens these partially recovered boundaries preferentially, as they have higher stored energy and are more susceptible to boundary migration, thereby producing the lower mean circularity and equiaxed fraction observed in L2. This mechanistic interpretation is consistent with the present data but would require controlled experiments to confirm.

L1–L3 and L2–L3 is consistent with the continuous DRX (CDRX) pathway previously proposed for FSP-processed aluminum alloys [12], in which this combination is consistent with the bimodal grain structure (BGS) reported in Al–Cu WAAM–FSP systems [29,30,31,32], wherein a majority fine-DRX population coexists with a minority coarser-grain population produced by grain growth during the extended post-peak dwell at elevated temperature. The optical grain area distributions in Figure 4 are right-skewed but do not clearly exhibit two distinct peaks; accordingly, BGS is proposed as a consistent interpretation rather than a directly demonstrated feature of the present data. Definitive confirmation would require EBSD imaging to resolve the two grain populations crystallographically, as a mixture model fit to a right-skewed grain area distribution from optical microscopy would be underdetermined without a priori knowledge of the modal diameters of the two populations. The pairwise Mann–Whitney U tests with Bonferroni correction return *p*_Bonf < 10^−16^ for the L1–L3 and L2–L3 contrasts, which is consistent with the morphological transition representing a genuine population-level shift in grain character rather than a sampling artifact, and is consistent with the continuous DRX (CDRX) pathway previously proposed for FSP-processed aluminum alloys [12], in which progressive subgrain rotation generates high-angle boundaries without a discontinuous nucleation event.

### 4.2. Thermal–Defect Coupling: A Cooling-Rate-Governed Consolidation Mechanism

The cooling rate–porosity coupling discussed in this section is established between L1 and L3 at a common 250× SEM magnification; L2 is excluded from the primary porosity comparison (Section 3.3), and the mechanistic interpretation that follows is therefore conditioned on the L1–L3 contrast and is advanced as a hypothesis pending expanded multi-layer sampling. The cooling rate values employed in the discussion below are derived from a single build series with no replicate thermal histories (Section 2.4); the mechanistic interpretation is conditional on the directional consistency of the L1–L3 cooling rate difference (3.95 °C/s, substantially exceeding the slope confidence intervals of ±0.4–0.6 °C/s reported in Section 2.4) and would benefit from replicate IR acquisition in future builds.

The earlier presented thermal and defect results reveal that Layer 3 carries 80% more porosity than Layer 1, yet records a lower peak FSP surface temperature (Figure 9 and Figure 10), which is a result at odds with the expectation that reduced thermal input eases defect formation. One confound deserves upfront acknowledgment: Layer 1 was stirred at 600 rpm, whereas Layers 2 and 3 were stirred at 1200 rpm (Table 5), so layer height and rotational speed co-varied and could not be fully separated. To assess the directional contribution of the rpm increase quantitatively, a first-order estimate of the frictional heat input can be derived from Q ≈ (2/3)μF_n_ωR_s_, which represents full-contact sliding integrated over a flat circular shoulder (SI units: F_n_ in N, ω in rad/s, R_s_ in m, Q in W), where μ is the friction coefficient (~0.3 for Al–steel tool contact), F_n_ is the axial plunge force (~3000–5000 N for this shoulder geometry), ω is the angular velocity, and R_s_ is the shoulder radius (0.009 m). At 600 rpm (ω = 62.8 rad/s) this yields Q ≈ 1.3–2.2 kW; at 1200 rpm (ω = 125.7 rad/s), Q ≈ 2.6–4.4 kW. The (2/3) prefactor applies equally to both rpm levels and does not affect the directional conclusion. Doubling the frictional heat input at higher build layers would, if anything, be expected to raise the post-peak temperature and accelerate subsequent cooling through a larger thermal gradient, which is the opposite of the observed pattern, in which L3 exhibits both the lowest peak temperature and the slowest post-peak cooling rate. This directional consistency strongly supports the interpretation that progressive substrate thermal buffering, rather than the rpm increase, governs L3’s anomalous thermal behavior. It should nonetheless be acknowledged that the rpm confound cannot be fully excluded as a secondary contributor and that controlled single-variable experiments (constant rpm, varying build height) constitute the definitive test. The following mechanistic interpretation is proposed and supported by the available evidence, with the caveat that direct subsurface temperature and pressure measurements were not performed in this study.

The cooling rate values employed in the discussion below are derived from a single build series with no replicate thermal histories (Section 2.4); the mechanistic interpretation is accordingly conditional on the directional consistency of the L1–L3 cooling rate difference (3.95 °C/s, substantially exceeding the slope confidence intervals reported in Section 2.4) and would benefit from replicate IR acquisition in future builds. The dominance of post-peak cooling rate over peak temperature can be rationalized as follows: Void closure under FSP is driven by plastic flow under the hydrostatic pressure of the rotating shoulder while the matrix remains within the solid-state deformation window (~300–450 °C in the stir zone). Once the tool has passed, additional closure can still occur through differential thermal contraction, provided that the contraction rate outruns stress relaxation in the surrounding matrix. The total time spent above the stress-relaxation-active temperature is therefore the dominant kinetic variable, and this duration is governed by the cooling rate rather than the peak temperature. For the temperature range observed here (peak T_pak_ = 195–263 °C at the surface), the three layers spent comparable times above the stress-relaxation-active threshold, but their post-peak cooling rates differed by a factor of approximately 1.3 between L1 and L3; the same kinetic argument leads to the prediction that the slower-cooling L3 retains greater residual porosity, which is consistent with the observed data. Strain rate, while relevant during the FSP pass itself, is largely controlled by the shoulder geometry and rpm settings, which are deliberately held constant within each rpm group; accordingly, strain rate is not expected to drive the observed L3 anomaly.

This interpretation is proposed tentatively and would require direct compositional and crystallographic characterization for definitive confirmation. In the context of void consolidation, the closure during FSP is driven mainly by the plastic flow of matrix material into pore interiors under the hydrostatic pressure and shear stress generated by the rotating shoulder and pin. This mechanism operates while the material remains within the solid-state deformation window, estimated at roughly 300–450 °C for Al 4043 based on published FSP literature for this alloy system. The IR surface temperatures reported here (peak values of 195–263 °C) are substantially lower than this deformation window, which represents a primary instrumentation limitation of the present study; as noted in Section 2.4, surface thermography cannot directly measure stir zone temperatures. This apparent contradiction is resolved by the well-established disparity between surface-measured and subsurface stir zone temperatures in FSP: the rotating tool generates intense frictional and plastic dissipation heat locally within the stir zone, where temperatures typically reach 0.6–0.9 Tm (solidus) and are expected to lie within the 300–450 °C deformation window, while the IR camera captures only the cooler workpiece surface remote from the tool–material interface. This surface–subsurface temperature disparity has been directly documented in FSW and FSP of aluminum alloys through embedded thermocouple and thermocouple-pin measurements: Tang et al. [33] reported stir zone temperatures 150–250 °C above surface measurements in FSW of 6061-T6 aluminum, and Gerlich et al. [34] confirmed analogous through-thickness temperature gradients in friction stir spot welding of aluminum alloys, with peak stir zone temperatures substantially exceeding surface IR readings in both cases. The internal stir zone temperatures were not directly measured in this study, as described in Section 2.4. Once the tool has passed, further closure can still occur as the workpiece contracts on cooling, provided that the contraction rate outruns stress relaxation. For L1 at −16.2 °C/s, that condition is met and differential contraction assists the final pore closure. Both cooling rate values are derived from a single build series with no replicate thermal histories, as stated in Section 2.4. The mechanistic interpretation rests on the directional consistency of the L1–L3 difference of 3.95 °C/s, which substantially exceeds the reported slope confidence intervals of ±0.4–0.6 °C/s. Replicate IR acquisition over a minimum of three independent build series is a priority for the follow-on experimental program described in Section 4.3.

This observation is morphologically consistent with the proposed interpretation. As noted in Section 3.2.5, EDS or EBSD mapping was not performed in this study, which means that the mechanistic interpretation of preferential void persistence near particle–matrix interfaces remains hypothesis-level until supported by compositional characterizations. EDS or EBSD mapping was not performed in this study, which prevents direct confirmation of particle phase identity and compositional gradients near pore–particle interfaces. With this caveat, the SEM evidence at 12,000×–20,000× is consistent with the proposed interpretation at the morphological level: the consistent spatial co-location of sub-micron pores with fragmented second-phase particles in Layer 2 is consistent with preferential void persistence or nucleation near particle–matrix interfaces. EDS/EBSD mapping is identified as the single highest-priority future experiment for this work.

The spatial heterogeneity of porosity within L3, wherein position P2 (right edge) exhibited 3.82% versus 1.57% at position P3 (left edge), is also consistent with this mechanism. One possible contributor is a lateral gradient in tool shoulder pressure across the bead width, though the tool deflection and local pressure distribution were not directly measured in this study, and this interpretation therefore remains tentative. This position-dependence indicates that tool pressure uniformity is required for consistent defect mitigation in multi-pass multi-layer UAMFSP builds.

Earlier studies on FSP-assisted WAAM reported related trends; the present data add a layer-resolved thermal explanation that advances the mechanistic understanding. Sun et al. [35] demonstrated that interlayer FSP in WAAM 2319 Al eliminates macroscopic porosity while redistributing Al_2_Cu precipitates, improving tensile strength and ductility; the present work indicates that macro-closure does not preclude micro-porosity persistence under unfavorable thermal conditions. Also, Zhou et al. [36] reported substantial strength gains (~30%) in Al–Cu–Sc WAAM + FSP walls; the results of the present study indicate that these improvements may be partially offset by increased upper layer porosity if the interlayer cooling dynamics are not optimized.

### 4.3. Implications for Process Optimization

The earlier presented thermal–defect coupling results provide a first mechanistic framework for understanding how post-peak cooling rate governs porosity evolution in multi-layer UAMFSP builds, with practical implications for process design. Based on the data, the explicit management of the post-peak cooling rate, rather than peak temperature alone, is identified as the primary requirement for achieving void consolidation quality in multi-layer UAMFSP builds. Several engineering strategies follow from this analysis [37].

First, active cooling immediately after tool retraction (within the critical first 10 s post-peak window captured by IR thermography in this study), for example through compressed air or forced convection, would increase the post-peak cooling rate in the upper layers and bring the L3 conditions closer to the more favorable L1 thermal environment. This active post-FSP cooling is distinct from the existing inter-pass cooling protocol (38–40 °C baseline before restarting deposition) and would operate on a much shorter timescale, targeting the immediate post-tool dwell period rather than the bulk interlayer cooldown. Second, substrate pre-cooling before the upper layer deposition step could reduce the thermal buffering effect by restoring a stronger conduction gradient. Third, tool parameter optimization for the upper layers, specifically employing a higher traverse speed or reduced rotational speed for L3, could shorten the post-peak dwell at elevated temperature [38,39]; however, a layer-specific optimization scheme that independently controls heat input and stirring intensity is warranted.

Several directions would strengthen this work and are recommended for future investigations. Thermocouple or embedded sensor measurements inside the stir zone would decouple the surface and subsurface thermal histories, thereby directly addressing the key temperature measurement limitation of the present study. EDS and EBSD mapping of the stir zone would simultaneously confirm particle phase identity, provide direct evidence of compositional gradients near pore–particle interfaces, and supply crystallographic texture data and grain orientation mapping to complement the optical circularity analysis presented here. Extended builds of five or more layers would clarify whether the porosity gradient continues to grow or reaches a steady state, and tensile and fatigue testing are needed to quantify the mechanical consequences of the 80% porosity increase in L3. In addition, residual stress measurements by X-ray or neutron diffraction would characterize the net stress state following SPD redistribution. Also, a controlled constant-parameter experimental study is currently underway to isolate the thermal contribution independently of the rotational speed variation, which is expected to provide definitive evidence for or against the cooling rate hypothesis proposed here.

### 4.4. Engineering Implications of the Layer-Resolved Porosity Gradient

**Figure 12:**
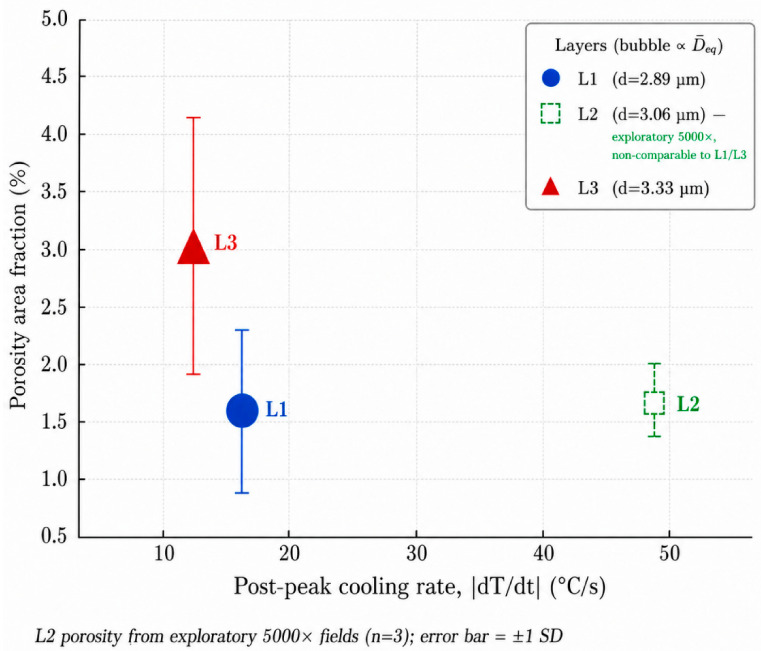
Process–structure–property bubble map for UAMFSP-processed Al 4043. Note: the L2 marker (dashed open outline) represents an exploratory 5000× SEM measurement (Supplementary Table S2) that is not directly comparable to the 250× L1 and L3 quantification and is included for positional context only; it does not constitute a confirmed data point in a monotonic trend. The L1 and L3 markers (filled) represent the directly comparable 250× SEM porosity quantification (Section 3.3). Bubble area scales with mean equivalent grain diameter $\bar{D_{eq}}$. Error bars = ±1 SD.

The L2 data point in Figure 12 is derived from exploratory 5000× SEM fields with a smaller field area than the 250× L1/L3 quantification (Supplementary Table S2) and is therefore presented for descriptive trend context only; it is rendered with a dashed open marker to flag this non-comparability. The layer-resolved porosity gradient documented in this study has direct consequences for the structural performance of UAMFSP-fabricated aluminum components. Residual porosity at the level of 2–3% area fraction has been reported to reduce the fatigue endurance limit by approximately 15–25% relative to fully dense aluminum, and to lower the ductile fracture strain by similar margins, owing to early stress concentration and void coalescence at pore sites. For the 80% layer-to-layer porosity increase reported here (L1 to L3), the upper layers of a multi-layer UAMFSP build are therefore expected to be the structurally critical region. The three-marker layout of Figure 12 does not constitute a confirmed monotonic three-layer trend; L1 and L3 represent the only statistically comparable porosity measurements in this study (250× SEM, identical field protocol), and the L2 marker is shown for positional reference only, not as an inferential data point, particularly under cyclic loading where pore-driven crack initiation governs life. The L1 and L3 markers in Figure 12 represent the directly comparable 250× porosity quantification, while the L2 marker is included for descriptive context only at a smaller field area (Supplementary Table S2, 5000× SEM) and is not directly comparable to L1 and L3. Figure 12 summarizes these process–structure–property relationships as a bubble map, plotting post-peak cooling rate against porosity area fraction with bubble area proportional to mean equivalent grain diameter.

From a qualification perspective, the layer dependence indicates that bulk porosity averages across the full wall cross-section may underestimate the local defect population in the upper layers; build-direction-resolved testing or layer-specific NDE acceptance criteria are accordingly recommended for any safety-critical UAMFSP application. This concern is particularly relevant for tall WAAM–FSP builds, where the cumulative thermal buffering effect documented here is expected to amplify the upper layer porosity gradient. Collectively, these implications motivate the engineering strategies described in Section 4.3 (active post-FSP cooling, substrate pre-cooling, and layer-specific tool parameter optimization) and the controlled mechanical test program identified as priority future work.

## 5. Conclusions

In this study, multi-layer Al 4043 walls fabricated through the UAMFSP were examined across their thermal, microstructural, and defect characteristics, employing IR thermography, optical grain morphology analysis, multi-scale SEM, and calibrated porosity quantification. The results are presented as a correlative mechanistic framework in which post-peak cooling rate is identified as a plausible controlling factor for porosity evolution in hybrid WAAM–FSP builds, with the caveat that complete causal isolation requires controlled single-variable experiments. Six principal findings emerge from this investigation:

1. The UAMFSP produces highly refined microstructures across all three build layers, with the mean equivalent grain diameter confirmed to be below 3.4 μm in every layer, thereby confirming effective DRX-driven grain refinement throughout the full build height. This remarkable degree of refinement is consistent with the well-established role of the FSP in promoting severe plastic deformation and dynamic recrystallization in aluminum alloys.
2. Grain morphology evolves non-monotonically with build height: Layer 2 displays the lowest mean circularity (0.569) and equiaxed fraction (25.5%), while Layer 3 achieves the highest values (0.645 and 36.1%). One-way ANOVA (F = 56.2, *p* = 5.15  ×  10^−25^) and Kruskal–Wallis (H = 121.3, *p* = 4.69 × 10^−27^) confirm that these interlayer differences are statistically significant. The non-monotonic pattern is attributed to layer-specific FSP thermomechanical conditions, differential reheating from subsequent deposition, and layer-resolved post-peak cooling rates, with the mechanistic interpretation requiring future controlled experiments to fully decouple these contributions. In a substantial fraction of the imaged fields, this co-location is observed, although secondary electron imaging alone does not establish this mechanism.
3. Multi-scale SEM imaging (250×–35,000×) resolved the second-phase particle landscape of UAMFSP-processed Al 4043: the Al–Si eutectic fragments exhibited a range of morphologies from elongated laths (AR ≈ 3–8) to partially spheroidized compact particles, reflecting incomplete spheroidization under single-pass FSP conditions. Sub-micron gas/shrinkage-type pores are spatially co-located with second-phase particles in a substantial fraction of the imaged fields. This co-location is consistent with preferential void persistence near particle–matrix interfaces, although secondary electron imaging alone does not establish this mechanism. Confirmation via EDS/EBSD compositional mapping is identified as a priority for future work.
4. The porosity area fraction was found to increase substantially from L1 (1.61 ± 0.75%) to L3 (2.90 ± 1.18%), representing an 80% increase, alongside a 107% increase in pore density (2068 to 4283 pores/mm^2^). This trend is counterintuitive given that L3 records a 26% lower peak FSP surface temperature than L1, which further identifies post-peak cooling rate as the governing thermal metric for void consolidation quality.
5. The cooling rate–porosity relationship reported here is established between two directly comparable layers (L1 and L3), and is therefore advanced as a hypothesis to be tested in future work, in which expanded sampling across all three layers at a common magnification is recommended. The cooling rate values that underpin this interpretation are derived from a single build series; replicate IR acquisition over multiple builds is identified as a priority for the controlled experimental study described in Section 4.3. The primary porosity comparison in this study is limited to L1 and L3, both quantified from calibrated 250× SEM fields (*n* = three fields per layer); the L2 estimate provided in Supplementary Table S2 was acquired at a different magnification and field area and is excluded from the primary statistical inference. Matched magnification porosity acquisition across all three layers in future builds will enable a regression-based test of the proposed cooling rate mechanism across the full build height. The cooling rate–porosity relationship reported here is established between two directly comparable layers (L1 and L3) at a common 250× SEM magnification, while the L2 porosity estimate provided in Supplementary Table S2 is descriptive (5000× field area; *n* = three fields) and not directly comparable to the L1/L3 dataset; the interpretation is therefore advanced as a hypothesis to be tested in future work in which expanded sampling across all three layers at a common magnification is recommended. The apparent contradiction between the higher porosity in L3 and its lower peak temperature is resolved by the post-peak cooling rate: L3 cools at only −12.3 °C/s versus −16.2 °C/s for L1, despite the lower peak temperature. The slower cooling of L3 reflects the cumulative substrate thermal buffering after three successive deposition–FSP cycles, which reduces the temperature gradient available for conduction cooling. This extended post-peak dwell at elevated temperature impairs void closure, supporting the interpretation that the post-peak cooling rate, rather than peak temperature, is a more informative thermal metric for defect consolidation quality in the present build configuration. The cooling rate values that underpin this interpretation are derived from a single build series with no replicate thermal histories (Section 2.4); replicate IR acquisition over a minimum of three independent build series is identified as a priority for the controlled experimental study described in Section 4.3, in which expanded porosity sampling across all three layers at a common 250× magnification is also recommended. Future work employing controlled single-variable experiments is needed to fully decouple these effects and elevate the present correlation to a causal mechanistic relationship.
6. The primary technological implication of this first mechanistic framework is that post-peak cooling rate warrants explicit management on a layer-by-layer basis in UAMFSP multi-layer builds. Active post-FSP cooling, substrate pre-cooling, or layer-specific traverse speed optimization represent practical engineering pathways that can be employed to reduce upper layer porosity while maintaining the grain refinement benefits established at lower layers. More broadly, the finding that the post-peak cooling rate governs defect consolidation quality is expected to be relevant to similar hybrid AM systems in which interlayer heat accumulation progressively modifies the solid-state post-processing thermal environment, suggesting that the present findings may have value beyond the specific UAMFSP configuration examined here; however, direct validation in other hybrid AM systems is needed before broader generalization.

## Figures and Tables

**Table 1:** Nominal chemical compositions (wt.%) of the AA6061 substrate and the ER4043 filler wire.

Material	Mg	Fe	Mn	Cr	Si	Cu	Zn	Ti	Al
AA6061 substrate	0.8–1.2	≤0.70	≤0.15	0.04–0.35	0.40–0.80	0.1–0.4	≤0.25	≤0.15	bal.
ER4043 filler wire	≤0.05	≤0.80	≤0.05	N/A	4.5–6.0	≤0.30	≤0.10	≤0.20	bal.

**Table 2:** Consolidated UAMFSP parameters per layer.

Parameter	L1	L2	L3
MIG voltage (V)	18	18	18
MIG current (A)	120	120	120
Travel speed (mm/min)	330	330	330
Wire stick-out (mm)	9	9	9
Argon flow rate (CFH)	27	27	27
FSP rotational speed (rpm)	600	1200	1200
FSP traverse speed (mm/min)	50	50	50
FSP plunge depth (mm)	0.2	0.2	0.2
Interlayer T target (°C)	38–40	38–40	38–40

**Table 3:** Grain morphology statistics for UAMFSP-processed Al 4043 (Layers 1–3).

Layer	N (Grains)	Ā (μm^2^)	σA (μm^2^)	$\bar{D_{eq}}$ (μm)	$\bar{C}$	$\bar{AR}$	EqFrac (%)
L1	2174	8.30	7.90	2.89	0.621	1.370	29.6
L2	2167	8.82	8.13	3.06	0.569	1.352	25.5
L3	6005	12.55	15.65	3.33	0.645	1.307	36.1

Note: Overbar = mean; σ = standard deviation; EqFrac = equiaxed grain fraction (C ≥ 0.65). AR (Aspect Ratio Proxy) = ratio of major to minor axis of the best-fit ellipse per grain as reported by MIPAR. L3 grain count (N = 6005) is ~2.8× larger than L1/L2 owing to greater image sampling area; L3 statistics are therefore more precise but the count difference does not reflect greater microstructural variability.

**Table 4:** Field-by-field and layer summary porosity quantification from calibrated 250× SEM images. Pore density computed from raw count per analyzed area. Summary rows show mean ± standard deviation across three fields.

Layer	Location	ROI Area (mm^2^)	Porosity (%)	Pore Density (Pores/mm^2^)
L1	P1: center	0.01140	2.45	2895
L1	P2: right edge	0.00697	1.35	2151
L1	P3: left edge	0.00950	1.03	1158
L3	P1: center	0.01406	3.30	3769
L3	P2: right edge	0.00695	3.82	5323
L3	P3: left edge	0.00692	1.57	3758
L1 (mean)	*n* = 3 fields	0.02787	1.61 ± 0.75	2068 ± 871
L3 (mean)	*n* = 3 fields	0.02793	2.90 ± 1.18	4283 ± 900

**Table 5:** IR-derived thermal metrics for each UAMFSP layer pass (ε = 0.95, 15 fps, surface T_max_). Cooling slope = linear regression of T_max_(t) over first 10 s post-peak.

Layer	Peak T_max_ (°C)	Heating Rate (°C/s)	Cooling Slope (°C/s)	Time to Peak (s)
L1	263.2	3.00	−16.23	61.1
L2	238.9	3.89	−46.88	40.9
L3	195.1	4.10	−12.28	28.1

## Data Availability

The raw data supporting the conclusions of this article will be made available by the authors on request.

## Supplementary Materials

The following supporting information can be downloaded at: https://www.mdpi.com/article/10.3390/ma19132922/s1.

## Abbreviations

The following abbreviations are used in this manuscript:

ANOVA	Analysis of Variance
AR	Aspect Ratio Proxy
BGS	bimodal grain structure
CDRX	Continuous Dynamic Recrystallization
CI	confidence interval
DED	Directed Energy Deposition
DRX	dynamic recrystallization
EBSD	Electron Backscatter Diffraction
EDS	Energy-Dispersive X-ray Spectroscopy
ER4043	Al–Si welding wire/filler alloy (AWS designation)
FSP	friction stir processing
FSW	friction stir welding
HV	Vickers Hardness
IR	infrared
KDE	Kernel Density Estimate
MIG	Metal Inert Gas
PAW	plasma arc welding
ROI	Region of Interest
SEI	secondary electron imaging
SEM	scanning electron microscopy
SPD	severe plastic deformation
TIG	Tungsten Inert Gas
UAMFSP	Unified Additive–Deformation Manufacturing Process
WAAM	wire arc additive manufacturing
