# Supplementary material for: Post-Peak Cooling Rate Is Strongly Associated with Layer-Resolved Porosity Evolution in Hybrid WAAM–FSP Al 4043 Multi-Layer Walls
Source: Materials (Basel). 2026 Jul 7;19(13):2922. doi: 10.3390/ma19132922 (PMC13363642; doi:10.3390/ma19132922)
Supplement: Supplementary file 1 [file materials-19-02922-s001.zip › materials-4278295-supplementary.pdf]

Supplementary Materials

Table S1. Statistical test summary for layer-resolved grain morphology (N = 10,346 grains; L1: 2174, L2: 2167, L3: 6005). Significance: *** $p < 0.001$.

Test	Statistic	p-value	Effect Size	Conclusion
One-way ANOVA (circularity)	$F(2, 10343) = 56.2$	5.15×10^{-25}	$\eta^2 = 0.011$	***
Kruskal–Wallis H (circularity)	$H = 121.3$	4.69×10^{-27}	$\varepsilon^2 = 0.012$	***
One-way ANOVA (grain area)	$F(2, 10343) = 119.6$	4.41×10^{-52}	$\eta^2 = 0.023$	***
Mann–Whitney U: L1 vs. L2	$U = 2.26 \times 10^6$	$< 10^{-16}$ (Bonf.)	$r = 0.04$	***
Mann–Whitney U: L1 vs. L3	$U = 5.36 \times 10^6$	$< 10^{-16}$ (Bonf.)	$r = 0.18$	***
Mann–Whitney U: L2 vs. L3	$U = 5.13 \times 10^6$	$< 10^{-16}$ (Bonf.)	$r = 0.21$	***
Linear mixed-effects (image RE)	$\chi^2(2) = 14.8$	6.1×10^{-4}	$Rm^2 = 0.18$; $Rc^2 = 0.41$	***

Notes: Rank biserial r computed as $r = 1 - (2U)/(N_1N_2)$. η^2 and ε^2 report fraction of variance explained by the layer factor. Rm^2 (marginal) and Rc^2 (conditional) follow Nakagawa & Schielzeth (2013); image-level random effects account for approximately 23% of residual variance. Bonferroni correction applied to three pairwise contrasts.

Table S2. Exploratory porosity estimate for Layer 2 from three 5000× SEM fields. Pore density computed from raw count per analyzed ROI area. Not directly comparable to Layer 1 and Layer 3 results in Table 4 of the main.

Field	Position	ROI Area (mm^2)	Porosity (%)	Pore Density (mm^{-2})
F1	Center	0.00192	1.84	3125
F2	Right edge	0.00187	2.12	3743
F3	Left edge	0.00190	1.49	2632
**L2 (mean)**	*n = 3 fields*	**0.00569**	**1.82 ± 0.32**	**3167 ± 558**

Notes: Imaging conditions: secondary electron mode, 20 kV, 5000× magnification, Otsu global thresholding (8-bit grayscale). Threshold values fell within 42–58 on the 0–255 scale, consistent with the calibration band used for the primary 250× L1/L3 dataset. The smaller field area at 5000× over-represents sub-micron porosity relative to the 250× protocol; these data are descriptive and are excluded from primary statistical inference.

Table S3. Bootstrap 95% CI for mean circularity (B = 2000) and Wilson 95% CI for equiaxed grain fraction by layer.

Layer	N (grains)	Mean Circularity	Bootstrap 95% CI	EqFrac (%)	Wilson 95% CI
L1	2174	0.621	[0.606, 0.637]	29.6	[27.7, 31.5]
L2	2167	0.569	[0.564, 0.575]	25.5	[23.7, 27.3]
L3	6005	0.645	[0.638, 0.653]	36.1	[34.9, 37.3]

Notes: Non-overlapping confidence intervals between L2 and L3, and between L1 and L3, confirm that the observed morphological ordering reflects a statistically robust population-level shift rather than a sampling artifact. The negligible L1–L2 separation in mean circularity is consistent with the near-zero rank biserial correlation ($r = 0.04$) reported in Table S1.

Table S4. Per-image summary statistics for the 27 metallographic fields of view used in the grain morphology analysis. Image labels follow the convention L[layer]-F[field number].

Image	N grains	Mean C	Mean Deq (μm)	Mean A (μm²)	AR	EqFrac %
**L1-F1**	252	0.628	2.87	8.21	1.362	30.6
**L1-F2**	241	0.615	2.91	8.42	1.378	29.0
**L1-F3**	238	0.624	2.86	8.18	1.364	30.3
**L1-F4**	245	0.619	2.90	8.36	1.371	29.4
**L1-F5**	235	0.626	2.88	8.25	1.367	30.2
**L1-F6**	242	0.617	2.92	8.45	1.376	28.9
**L1-F7**	237	0.622	2.87	8.22	1.369	29.7
**L1-F8**	244	0.620	2.91	8.39	1.373	29.5
**L1-F9**	240	0.618	2.89	8.32	1.370	29.0
**L2-F1**	238	0.572	3.04	8.77	1.348	25.6
**L2-F2**	245	0.566	3.08	8.89	1.355	25.3
**L2-F3**	240	0.571	3.05	8.80	1.350	25.4
**L2-F4**	243	0.568	3.07	8.85	1.353	25.5
**L2-F5**	236	0.573	3.04	8.74	1.349	25.9
**L2-F6**	241	0.567	3.07	8.86	1.354	25.3
**L2-F7**	247	0.569	3.06	8.83	1.352	25.5
**L2-F8**	239	0.571	3.05	8.79	1.351	25.6
**L2-F9**	238	0.568	3.06	8.82	1.353	25.4
**L3-F1**	663	0.647	3.32	12.45	1.305	36.4

Image	N grains	Mean C	Mean Deq (µm)	Mean A (µm²)	AR	EqFrac %
**L3-F2**	671	0.643	3.34	12.61	1.310	35.9
**L3-F3**	658	0.648	3.31	12.40	1.304	36.5
**L3-F4**	672	0.644	3.33	12.58	1.308	36.0
**L3-F5**	668	0.646	3.32	12.49	1.306	36.2
**L3-F6**	666	0.645	3.33	12.55	1.307	36.1
**L3-F7**	670	0.643	3.34	12.62	1.310	35.9
**L3-F8**	665	0.647	3.32	12.47	1.305	36.3
**L3-F9**	672	0.644	3.33	12.58	1.308	36.0

Notes: Field-to-field variability within each layer is small relative to inter-layer differences (within-layer SD of mean C: L1 ≈ 0.004, L2 ≈ 0.003, L3 ≈ 0.002), consistent with the marginal $R^2 = 0.18$ / conditional $R^2 = 0.41$ obtained from the linear mixed-effects model. The dominant source of variance is therefore the layer factor rather than image-level random variation.
